# Influence of dissolved organic matter in natural and simulated water on the photochemical decomposition of butylparaben

**DOI:** 10.1186/s40201-015-0185-z

**Published:** 2015-04-14

**Authors:** Marta Gmurek, Magdalena Olak-Kucharczyk, Stanisław Ledakowicz

**Affiliations:** Faculty of Process and Environmental Engineering, Department of Bioprocess Engineering, Lodz University of Technology, Wolczanska 213, 90-924 Lodz, Poland

**Keywords:** Photochemical degradation, Natural and synthetic water, Butylparaben, Humic acid, Hydroxyl radicals, Singlet oxygen

## Abstract

**Background:**

In the last few decades the quality of natural water has often deteriorated as a variety of novel pollutants have contaminated rivers and lakes. Trace amounts of some man-made chemicals can be hazardous to plants, animals as well as human health as carcinogens, mutagens or endocrine disruptors**.** Light radiation may help in its decomposition, aided by naturally occurring colored organic compounds (humic substances) in the water**.** The aim of these studies was to check the influence of presence of organic and inorganic matter on the removal of endocrine disrupting compound - butylparaben (BP) from water.

**Methods:**

Photochemical decomposition of BP in aqueous solution using: photolysis by ultraviolet-C (UVC) and visible (VIS) irradiation, advanced oxidation in H_2_O_2_/UV system and photosensitized oxidation was examined. The degradation processes were carried out in different type of water matrix: natural water from Sulejow Reservoir, simulated natural water with humic acids and buffered solution.

**Results:**

The presence of dissolved organic matter in water did not influence much on UVC photolysis and increases only about 8% of BP depletion rate in H_2_O_2_/UV system. While during visible light photolysis and photosensitized oxidation the addition of natural water matrix causes the acceleration of reaction rate by 16% and 36%, respectively. Moreover BP degradation proceeds via singlet oxygen generated from humic substances.

**Conclusions:**

Butylparaben undergoes both direct and indirect photodegradation in aqueous solution under UVC and visible radiation. The efficiency of the H_2_O_2_/UV process, photolysis as well as photosensitized oxidation processes is strongly dependent on composition of the water.

## Background

In the last few decades the quality of natural water has often worsened owing to contamination with man-made trace organic chemicals. These are sometimes carcinogens, mutagens or endocrine disruptors. Such substances are often not removed in traditional wastewater treatment, are not easily biodegradable, and may accumulate in organisms [1-4]. Polluted water is a threat to human and environmental health. Contaminations have also influence on the condition and population sizes of animals and plants in natural waters. Therefore, the discovery of efficient degradation methods for these pollutants is of wide interest [5-8].

Photochemical degradation of these substances has been attempted and may be influenced by the array of dissolved organic matter (DOM), e.g., humic and fulvic acids, and by nitrate as well as other inorganic ions [9]. Hydrogen peroxide is also present in natural water, and generated mostly by solar radiation and microbial processes with concentrations in the range 10^−6^ - 10^−5^ M [10]. Reactive oxygen species (ROS) are also generated in the environment through photochemical processes and may be crucial to photochemical degradation. They include singlet oxygen (^1^O_2_), hydroxyl radical (^•^OH), superoxide radicals (HO_2_^-•^/O_2_^-•^), and peroxyl radical (^•^OOR) as well as non-ROS transients, e.g., carbon radicals (CH_3_^•^) and triplet exited states of DOM (^3^DOM*) [11-15]. The production of these diverse array of reactive species is driven primarily by abiotic photochemical reactions involving naturally occurring organic and sometimes inorganic coloured substances (chromophores) [16].

The absorption of solar radiation between UVB and visible wavelengths by DOM in natural waters initiates a series of complex photochemical reactions that improve water -purification. The chromophores present within DOM become excited from their singlet ground state to their first excited singlet stated (^1^DOM^*^). Upon absorption of light, undergo intersystem crossing (ISC) to form their triplet excited state (^3^DOM^*^), and then interact with molecular oxygen to form either singlet oxygen (^1^O_2_) or superoxide (O_2_^–•^). Hydroxyl radicals (^•^OH), another common reactive oxygen species, can be formed from the interaction of water with ^3^DOM^*^, the photolysis of hydrogen peroxide (H_2_O_2_), or the photolysis of nitrate (NO_3_^−^) [16].

**Figure 1:**
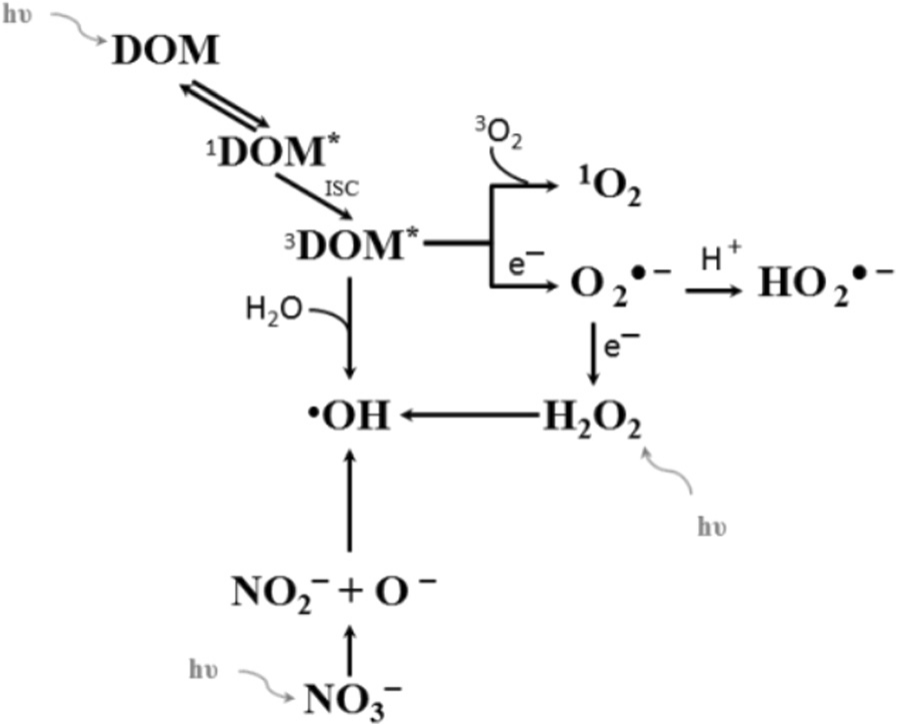
The pathways of photochemical generation of reactive oxygen species in natural water.

As a result of the reactions described in Figure 1., concentrations of reactive oxygen species in natural waters have been detected in the range between 10^−15^ - 5 × 10^−13^ M, 10^−15^ - 10^−12^ M, 10^−9^ - 10^−8^ M, 10^-18^- 2 × 10^−16^ M, for ^3^DOM^*^, ^1^O_2_, O_2_^–•^, and ^•^OH, respectively [16]. The ROS can help to purify the aquatic environment of bioactive pollutants derived from human activities e.g. pharmaceuticals and personal care products, or, in some instances, convert them to less toxic substances. The reaction sequences followed by well-established concepts of direct and sensitized photooxidations, also potentially coupled to thermal autooxidation processes [16]. Although the role of reactive oxygen species in water purification is known, the mechanism of these processes is still not entirely clear. This work examined degradation of an endocrine disrupting compound (butylparaben (BP)) in aqueous solution using photolysis by Ultraviolet C and visible irradiation, advanced oxidation in a H_2_O_2_/UV system and photosensitized oxidation (POx). Degradation was carried out in different waters matrix: natural water from Sulejow Reservoir, simulated natural water with humic acids (HA), and buffered solution. The aim was to establish the influence of organic and inorganic matter on the removal of butylparaben from the water.

## Materials and methods

### Chemicals and reagents

Butylparaben (BP) (>99%, Table 1), synthetic humic acid (HA, technical ), tert-butanol and sodium azide (99%) were purchased from Fluka. Hydrogen peroxide (30%), disodium phosphate (Na_2_HPO_4_), monopotassium phosphate (KH_2_PO_4_) and potassium nitrate (KNO_3_) all p.a. were purchased from POCh, Poland. Meso-tetra(4-sulphonatophenyl)porphin (TPPS_4_) was bought from Sigma-Aldrich. The element composition of Fluka humic acid (HA, cat. no. 53680) has been reported to contain 48.36% of C, 26.91% of O, 4.24% of H, 0.78%of N and 0.78% of S [17]. The atomic ratios described by Rodrigues et. al. [17], are 1.04 (H/C), 0.42 (O/C), and 0.012 (N/C) which are within the values reported in the literature for soil HA, with the exception of the N/C ratio. All chemicals were used as received.

**Table 1:** **Physicochemical properties of butylparaben** [19,20,23]

	CAS No.	94-26-8
	molecular formula	C_11_H_14_O_3_
	molar mass	194.23
	solubility in water	0.77 μM at 25°C; 7.5 μM at 80°C
	log *K*_OW_	3.5
	pKa	8.24

### Model solutions and natural water samples

Aqueous solutions of butylparaben were made by diluting it into: natural water from Sulejow Reservoir (RW), simulated natural water with humic acids (SN1^pH7^ and SN1^pH9^) or with nitrate ions (SN2) and buffered solution (BS). The concentration of butylparaben was 8 × 10^−5^ M. In experiments using the H_2_O_2_/UV system, an optimal concentration of hydrogen peroxide of 0.01 M was used. In buffer solution experiments with an xenon arc lamp (XBO), TPPS_4_ was used as a photosensitizer at an optimal concentration of 2 × 10^−5^ M [18]. Buffer solutions were prepared from deionized water, purified using a Millipore Milli-Q Plus System (>18.2 MΩ). The pH of the deionized solution was adjusted by adding phosphate buffer: Na_2_HPO_4_-KH_2_PO_4_. For more details, see [18,19]. Synthetic solutions with organic matter (SN1^pH7^ and SN1^pH9^) and nitrate (SN2) were made by diluting humic acids and potassium nitrate, respectively.

Natural water was collected in summer from the Sulejow Reservoir, situated in central Poland in the middle course of the Pilica River (latitude: 51°27.2′, longitude: 19°59.2′, area:27 km^2^). Water for chemical analyses was filtered through Whatman GF/F (0.45 μm) filters and stored at 4°C before use. The pH of the reservoir water was 8.11, and its concentration of dissolved organic carbon (DOC) was 6.85 mg L^−1^. The concentrations of major ions in the reservoir water were: Ca^2+^ (34.02 mg L^−1^), K^+^ (9.79 mg L^−1^), Na^+^ (6.51 mg L^−1^), SO_4_^2−^(21.73 mg L^−1^), Cl^−^ (12.34 mg L^−1^), NO_3_^−^ (0.46 mg L^−1^). Mg^2+^, NH_4_^+^, Li^+^, F^−^, NO_2_^−^, Br^−^, PO_4_^3−^ ions were detected at concentrations of: 0.969 mg L^−1^, 0.044 mg L^−1^, 0.003 mg L^−1^, 0.112 mg L^−1^, 0.025 mg L^−1^, 0.007 mg L^−1^, 0.003 mg L^−1^, respectively.

The buffer solutions (BS) were prepared at three different pH values. BS1 had similar pH to the reservoir water (RW). BS2 was used at pH 7 to compare the influences of additives in the humic acid (SN1^pH7^) and nitrate (SN2) treatments. Experiments with humic acids and visible-light irradiation were done at pH 9 (BS3 and SN1^pH9^).

### Photodegradation experiments

The photodegradation experiments were conducted using UVC and xenon arc lamps as sources of light. The UVC direct photolysis and degradation in a H_2_O_2_/UV system were carried out in a rotating device with quartz test tubes (10 mL), placed between two exposure panels, each of them consisting of three 7.2 W lamps. Low pressure lamps (LP, Luzchem) emitting mainly at a wavelength λ = 254 nm were used. For more details, see [19,20]. Photon flux rate entering the reaction space, calculated on the basis of actinometric experiments with uranyl oxalate [21] was equaled 1 × 10^−5^ einstein L^−1^ s^−1^.

Photosensitizing oxidation experiments were carried out with an immersion xenon arc lamp located in a quartz well with cooling jacket. A 100 W xenon arc lamp (XBO, 100 W, Osram) served as the light source to simulate solar light irradiation. The lamp was surrounded by five plate reactors (volume 0.01 L), each placed 11 cm from the light source. Each reactor consisted of two glass plates (10 cm × 6 cm) bound with silicone seal such that the distance between the inner surfaces of the plates was 0.3 cm. The tested solutions were aerated and agitated by gas bubbling. The quantity of absorbed photons was calculated using Reinecke’s actinometer for wavelengths ranging from 310 to 770 nm [22], and was 3.24 × 10^−4^ einstein L^−1^ s^−1^. The 2 × 10^−5^ M solution of TPPS_4_ absorbed 3.77 × 10^−5^ einstein L^−1^ s^−1^*.*

### Analysis

The butylparaben decay rate was monitored by high pressure liquid chromatography (HPLC) coupled with UV detection using a Waters apparatus. Analysis was performed with a Waters Nova-Pak C18 column (3.9 mm × 150 mm) using a mobile phase consisting of a degassed mixture (70/30, v/v %) of methanol and acidified water (0.01% orthophosphoric acid) at a constant flow of 1 mL min^−1^. The detailed description of this analysis of BP is reported elsewhere [19,23]. The spectrophotometric analysis was performed on a Unicam UV 300 spectrophotometer. The dissolved organic carbon measurements were performed on a HACH IL 550TOC-TN apparatus. The ion concentrations were determined by an ion chromatograph (Dionex model ICS) on an IonPac CS18 (for cations) and an IonPac AS18 (for anions).

The performed experiments allowed us to estimate the extent of direct reaction of butylparaben with hydrogen peroxide in the absence of radiation, (“dark reaction”).

Results indicated an insignificant role of the direct reaction of studied compounds with H_2_O_2_. The blank reaction was carried out to investigate the hydrolysis of BP. The experiments showed no decomposition of the investigated compound in the dark after 12 h, much longer time than used during the photodegradation.

Experiments were performed in duplicate to assure accurate data acquisition.

## Results and discussion

**Figure 2:**
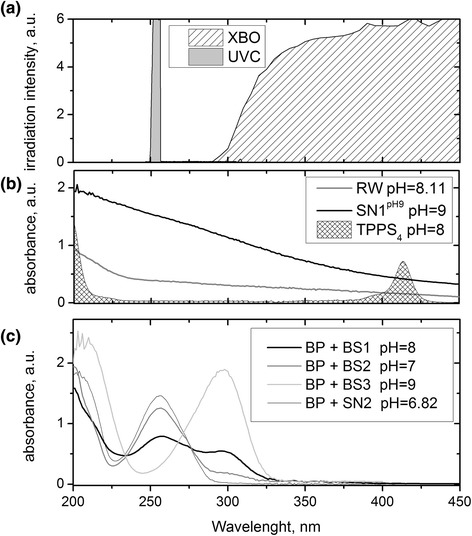
Emission spectra of lamps used in experiments **(a)**, absorption spectra of selected waters and TPPS_4_ **(b)** (C_TPPS4_ = 5 × 10^−6^ M, C_HA_ = 50 mg L^−1^) and BP solutions in different water matrix **(c)** (C_BP_ = 8 × 10^−5^ M).

For studies on photochemical degradation of butylparaben, the comparison of BP absorbance spectra in different reaction solutions with the emission spectra of the UVC and xenon arc (XBO) lamps were performed (Figure 2). Butylparaben in neutral solutions such as the humic acid and nitrate treatments, absorbs light mainly at low wavelengths up to 300 nm so its degradation by direct photolysis is expected to be higher using UVC systems. The degradation of butylparaben occurs by direct photolysis also in reservoir water (RW) and BS1, due to overlapping of the absorption spectrum of butylparaben with UVC and xenon arc lamps. In alkaline solution the direct photolysis of BP is likely as a results of occurring with xenon arc (XBO) irradiation.

The substances commonly found in natural waters, both organic and inorganic, may affect the photodegradation process of pollutants e.g. Cl^−^ ions accelerate the photodegradation of selected antibiotics under UV-Visible irradiattion (λ > 200 nm), whereas simulated solar irradiation (λ > 290 nm) was incapable of antibiotics photodecomposition, irrespective of Cl^−^ [24].

**Figure 3:**
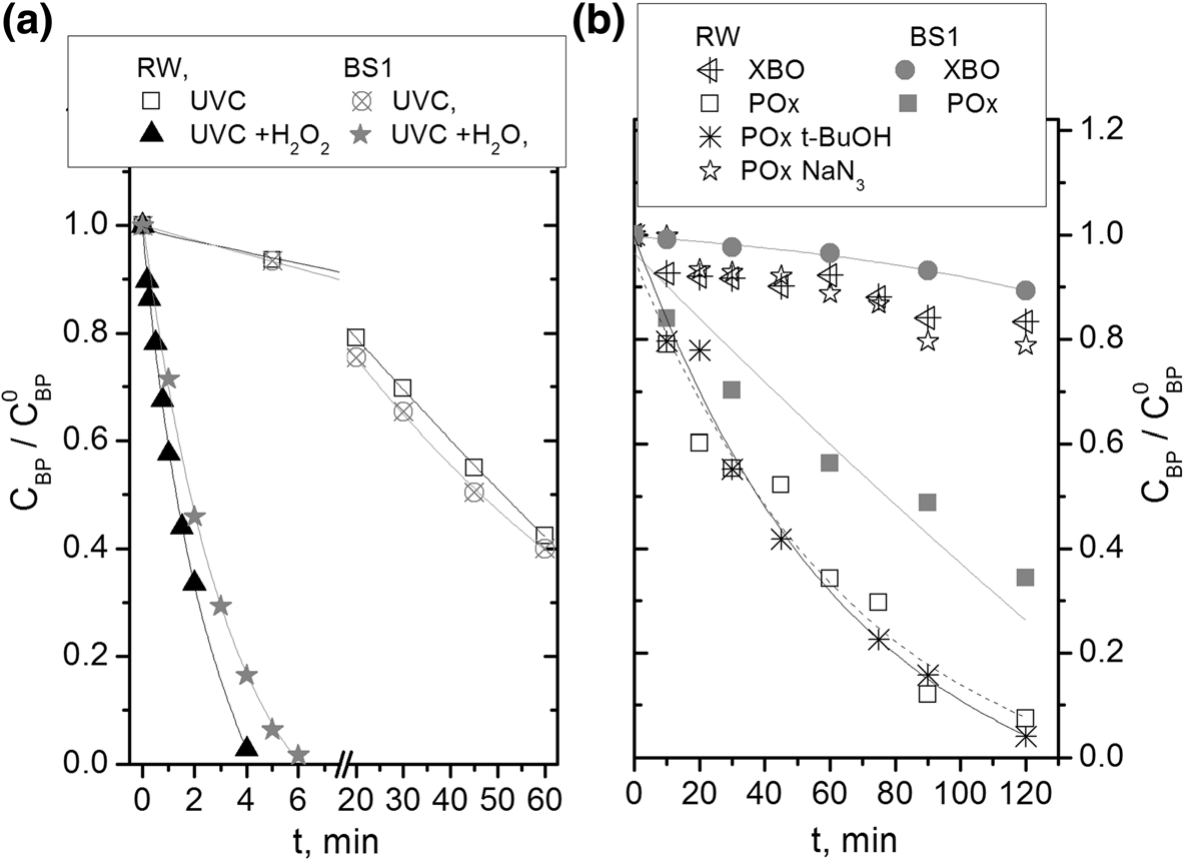
Changes of relative BP concentrations versus irradiation time during: UVC irradiation, and H_2_O_2_/UVC degradation **(a)**; VIS irradiation and photosensitized oxidation process (POx) **(b)** in natural water (pH = 8.11) and buffered solution (pH = 8.0) (E_0_^UV^ = 1 × 10^−5^ einstein L^−1^ s^−1^, *E*_0_^VIS^ = 3.24 × 10^−4^ einstein L^−1^ s^−1^, C_BP_ = 8 × 10^−5^ M, C_H2O2_ = 0.02 M, C_TPPS4_ = 2 × 10^−5^ M, C_t-BuOH_ = 0.1 M, C_NaN3_ = 0.02 M).

Figure 3 shows the degradation of butyparaben in water collected from Sulejow Reservoir (RW). UVC photolysis of butylparaben proceeded slightly slower (about 6%) (Figure 3a) in natural water compared with photolysis in buffered solution (BS1), whereas, the advanced oxidation of butylparaben in a H_2_O_2_/UV system was about 18% (Figure 3a) faster in reservoir water than in BS1. Butylparaben degradation in a H_2_O_2_/UV system can take place through direct photolysis and reaction with hydroxyl radicals generated during hydrogen peroxide photodecomposition. Addition of H_2_O_2_ to the irradiated RW and BS1 shortened the BP degradation by about 40 and 30 times, respectively.

Preliminary examinations using a xenon arc lamp showed that during 2 h of irradiation of buffered solution without photosensitizer the same degree of butylparaben degradation was achieved as in an experiment without irradiation. This means that direct photolysis was not occurring, but decrease of butylparaben concentration during both experiments was a result of the adsorption of butylparaben on silicone tube in the reactor. After 2 h of irradiation of natural water without TPPS_4_ the BP concentration decreased by 7% in comparison with experiments in buffered solutions (Figure 3b). Moreover in the dark, a decrease of butylparaben concentration was not observed if natural water was used. It is therefore possible that butylparaben decomposition occurs probably other than by adsorption. It is known that anions such as chloride, sulphate or carbonate have effects upon adsorption processes whilst phosphate and bicarbonate in the medium reduce the adsorption capacity [25,26].

Photolysis of organic compounds in water could be changed by the presence of naturally occurring organic and inorganic matter. Increasing the degree of butylparaben reduction by visible-light irradiation is presumably due to the natural dissolved constituents: humic acids, nitrate and chloride ions, where a strong influence on photolysis has been demonstrated [24,27]. Depending on quality and composition of water these elements can act as photosensitizers or conversely may exhibit quenching and scavenging effects [28,29].

Experiments using water from Sulejow reservoir showed that butylparaben concentration decreased by 93% after 2 h exposure in the reaction solution containing TPPS_4_ to visible radiation.whilst in buffer solutions, butylparaben concentration fell by 66%. The large difference in the butylparaben decomposition in these experiments may be caused by reactions involving components of the Sulejow water, such as dissolved organic matter. Dissolved organic material, by absorbing light can generate reactive oxygen species [30,31], which increase the rate of butylparaben decay. The used of a free radical scavenger – *tert*-butanol (*t*-BuOH), reduced butylparaben decomposition by hydroxyl radicals (Figure 3b). Sodium azide, a quencher of singlet oxygen, confirmed the role of singlet oxygen in the reaction mechanism. It can therefore be assumed that the DOM found in natural water, which resulted in increased reaction rate, has the ability to generate singlet oxygen. Humic substances present in low concentrations, can lead to photosensitized oxidation in the aqueous environment [32,33]. The differences in the effects of direct photolysis were observed, probably also due to the compounds contained in the natural water. However, experiments carried out with sodium azide resulted in inhibition of degradation to the level of that of direct photolysis, and *t*-BuOH did not affect the reaction. But the acceleration of the direct photolysis compared with reactions in buffered solution was not related to hydroxyl radicals or singlet oxygen, but probably to the presence of organic radicals.

**Figure 4:**
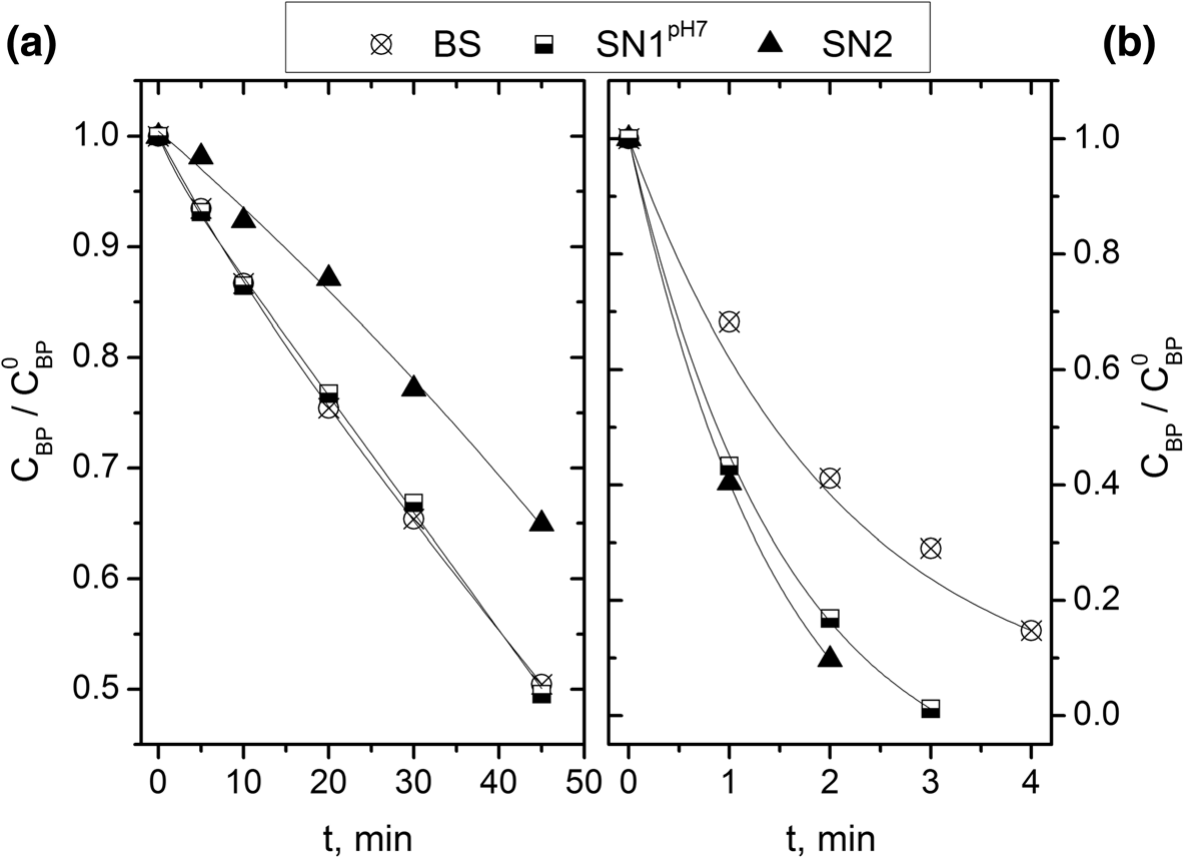
Changes of relative BP concentrations versus irradiation time upon degradation using UVC **(a)** and in H_2_O_2_/UVC system **(b)** in simulated natural water and buffered solution (SN1^pH7^ pH = 6.47; SN2 pH = 6.82, BS2 pH = 7, E_0_^UV^ = 1 × 10^−5^ einstein L^−1^ s^−1^, C_BP_ = 8 × 10^−5^ M, C_H2O2_ = 0.01 M, C_HA_ = 4.4 mg L^−1^, C_NO3-_ = 4.0 mg L^−1^).

The influences of additives on UVC photolysis as well as H_2_O_2_/UV system were investigated. The presence of humic acid (SN1^pH7^) in reaction solution upon UVC irradiation did not influence on butylparaben degradation, whilst the nitrate ions (SN2) inhibited BP decay (Figure 4a). Studies by Ge et al. [24], have shown that under UV-Visible irradiation (*λ* > 200 nm), NO_3_^−^ ions are able to inhibit to some extent the photodegradation rates, mainly due to their photoshielding effects. Humic acids can behave in irradiated solution as sensitizers, light filters and quenchers of free radicals, and therefore their show distinct effects on photodegradation of pollutants under UV irradiation (*λ* < 300 nm) and simulated sunlight [27]. Humic acids absorb UVC radiation (Figure 2b), which should slow down the butylparaben degradation. However, studies have shown that these compounds did not undergo photolysis at a wavelength of 254 nm [34]. Photoexcitation of humic acids may lead to the generation of reactive oxygen species resulting in the intensification of the degradation process, but at the same time humic acids can quench reactive oxygen species, so their effect on butylparaben photolysis may be negligible.

In the case of the H_2_O_2_/UV system, the presence of humic acid (SN1^pH7^) and nitrate ions (SN2) accelerated degradation of butylparaben by about 25% and 30%, respectively (Figure 4b). The increased decay rate of organic compounds in the presence of H_2_O_2_ and NO_3_^−^ was also observed by Li et al. [35] during simulated sunlight irradiation.

**Figure 5:**
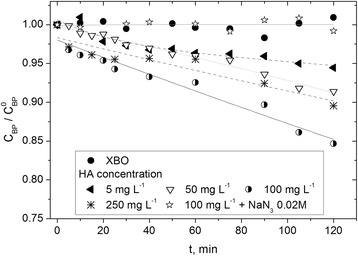
Changes of relative BP concentrations versus irradiation time upon degradation using visible light in simulated natural water (pH = 9, *E*_0_ = 3.24 × 10^−4^ einstein L^−1^ s^−1^, C_BP_ = 8 × 10^−5^ M, C_Na N3_ = 0.02 M).

The effects of humic acids in visible light on the degradation of butylparaben were examined (Figure 5) in a synthetic alkaline water (pH 9; BS3 and SN1^pH9^). To avoid the adsorption of butylparaben, the silicone tubes from the reactor construction were removed. The concentrations of humic acids (HA) were in the range: 5–250 mg L^−1^. As can be seen in Figure 5, BP photolysis is depended on the humic acid concentration. At first, increasing humic acid concentrations resulted in acceleration of initial rate of butylparaben decay, with the highest rate at 100 mg L^−1^. Further increase of humic acid concentrations reduced butylparaben decay primarily due to their light screening effect [36]. This process caused a decrease in efficiency of reactive oxygen species formation and butylparaben decomposition rate. It has been previously shown (Figure 1) that humic acids contain chromophores capable of sensitizing the generation of singlet oxygen. Therefore the excitation of humic acids leads to production of singlet oxygen. The experiments with azide anions, used as a singlet oxygen quencher, have indicated that singlet oxygen plays a dominant role in photodegradation of butylparaben, sensitized by humic substances. In summary, the photolysis rate of butylparaben can be enhanced by humic substances at low concentrations but could be inhibited at higher concentrations.

**Figure 6:**
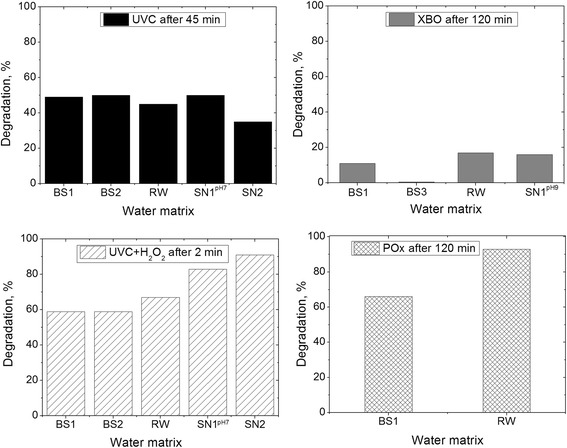
Influence of dissolved organic and inorganic matter in natural and simulated water on the BP photochemical degradation processes.

As can be seen from Figure 6, efficiency of the H_2_O_2_/UV, direct photolysis as well as photosensitized oxidation processes is strongly dependent on composition of the water.

It can be noticed that, the impact of dissolved organic and inorganic matter on the photochemical processes is related to light sources. Photolysis under xenon arc (XBO) radiation, and photosensitized oxidation in the presence of TPPS_4_ was fastest in reservoir water.

## Conclusions

Butylparaben undergoes both direct and indirect photodegradation in aqueous solution under UVC and visible radiation. The presence of dissolved organic matter in water did not influence on UVC photolysis and increases only about 8% of BP depletion rate in H_2_O_2_/UV system. While during visible light photolysis and photosensitized oxidation the addition of natural water matrix causes the acceleration of reaction rate by 16% and 36%, respectively.

The results indicate that butylparaben degradation proceeds via singlet oxygen, generated from humic substances. The presence of NO_3_^−^ lead to the highest butylparaben degradation rate in the H_2_O_2_/UV system. In the case of UVC light, only slight influences of water composition were observed. It could be shown that the water matrix plays an important role on the efficiency of photodegradation processes. However, the obtained results enrich the existing knowledge of the photosensitized oxidation as well as advanced oxidation processes in natural waters.

Although, the H_2_O_2_/UV system gave the fastest degradation of butylparaben ,the possibility of using sunlight in the photosensitized oxidation makes this method more attractive from a practical and economical point of view in the treatment of drinking water.

## Abbreviations

DOM: Dissolved organic substances
ROS: Reactive oxygen species
HA: Humic acids
O_2_: Singlet oxygen
OH: Hydroxyl radical
POx: Photosensitized oxidation
BP: Butylparaben
TPPS_4_: Meso**-**tetra(4**-**sulphonatophenyl)porphin
RW: Natural water from Sulejow Reservoir
SN1^pH7^: Simulated natural water with humic acids at pH 7
SN1^pH9^: Simulated natural water with humic acids at pH 9
SN2: Simulated natural water with nitrate ions
BS1: Buffered solution at pH 8
BS2: Buffered solution at pH 7
BS3: Buffered solution at pH 9
XBO: Xenon arc lamp
